# Potential Responses of Vascular Plants from the Pristine “Lost World” of the Neotropical Guayana Highlands to Global Warming: Review and New Perspectives

**DOI:** 10.3389/fpls.2017.00081

**Published:** 2017-01-25

**Authors:** Valentí Rull, Teresa Vegas-Vilarrúbia

**Affiliations:** ^1^Institute of Earth Sciences Jaume Almera (ICTJA-CSIC)Barcelona, Spain; ^2^Department of Evolutionary Biology, Ecology and Environmental Sciences, Universitat de BarcelonaBarcelona, Spain

**Keywords:** global warming, plant responses, acclimation, adaption, migration, habitat loss, extinction

## Abstract

The neotropical Guayana Highlands (GH) are one of the few remaining pristine environments on Earth, and they host amazing biodiversity with a high degree endemism, especially among vascular plants. Despite the lack of direct human disturbance, GH plants and their communities are threatened with extinction from habitat loss due to global warming (GW). Geographic information systems simulations involving the entire known vascular GH flora (>2430 species) predict potential GW-driven extinctions on the order of 80% by the end of this century, including nearly half of the endemic species. These estimates and the assessment of an environmental impact value for each species led to the hierarchization of plants by their risk of habitat loss and the definition of priority conservation categories. However, the predictions assume that all species will respond to GW by migrating upward and at equal rates, which is unlikely, so current estimates should be considered preliminary and incomplete (although they represent the best that can be done with the existing information). Other potential environmental forcings (i.e., precipitation shifts, an increase in the atmospheric CO_2_ concentration) and idiosyncratic plant responses (i.e., resistance, phenotypic acclimation, rapid evolution) should also be considered, so detailed eco-physiological studies of the more threatened species are urgently needed. The main obstacles to developing such studies are the remoteness and inaccessibility of the GH and, especially, the difficulty in obtaining official permits for fieldwork.

## Introduction

While pristine biomes and ecosystems are not subject to direct human pressure, they can be indirectly affected by anthropogenic forcing as expressed through ongoing global change, especially global warming (GW). The potential influence of GW on ecosystem composition, structure and function in pristine environments has been considered to be a novel and somewhat “unexpected” element that should be accounted for in conservation programs (Rull and Vegas-Vilarrúbia, 2006). Ecological and evolutionary studies of pristine areas are not only useful for discovering and protecting potentially endangered endemic and often unique species but also for unraveling the origin of biodiversity and its patterns on Earth. Additionally, such research increases the understanding of the long-term ecological functioning of ecosystems that are only subject to natural forcing. Untouched biomes are very rare, and the eventual consequences of GW could make them disappear. Therefore, it is urgently necessary to concentrate efforts on their study and conservation.

The neotropical Guayana Highlands (GH), the Lost World of Arthur Conan Doyle (1912), are so remote and inaccessible that they have remained virtually untouched until now. The GH have been considered a natural laboratory for addressing important and long-standing issues in neotropical ecology, evolution, and biogeography (Rull, 2010). The potential consequences of GW on GH vascular plants have been estimated given the 21st-century warming projected by the IPCC (Nogué et al., 2009; Safont et al., 2012; Vegas-Vilarrúbia et al., 2012). Emphasis has been placed on upward migration and eventual extinction by habitat loss, but other aspects such as the adaptation and acclimation potential of plants have not been fully explored. This paper reviews the results of the research that has been performed to date and provides perspectives on future research into the less developed aspects, acclimation and adaptation, and the potential genetic and ecological mechanisms that are involved. Studies of the potential consequences of GW for untouched biomes and ecosystems are uncommon, and the GH could serve as a pilot survey.

## The Guayana Highlands

**FIGURE 1:**
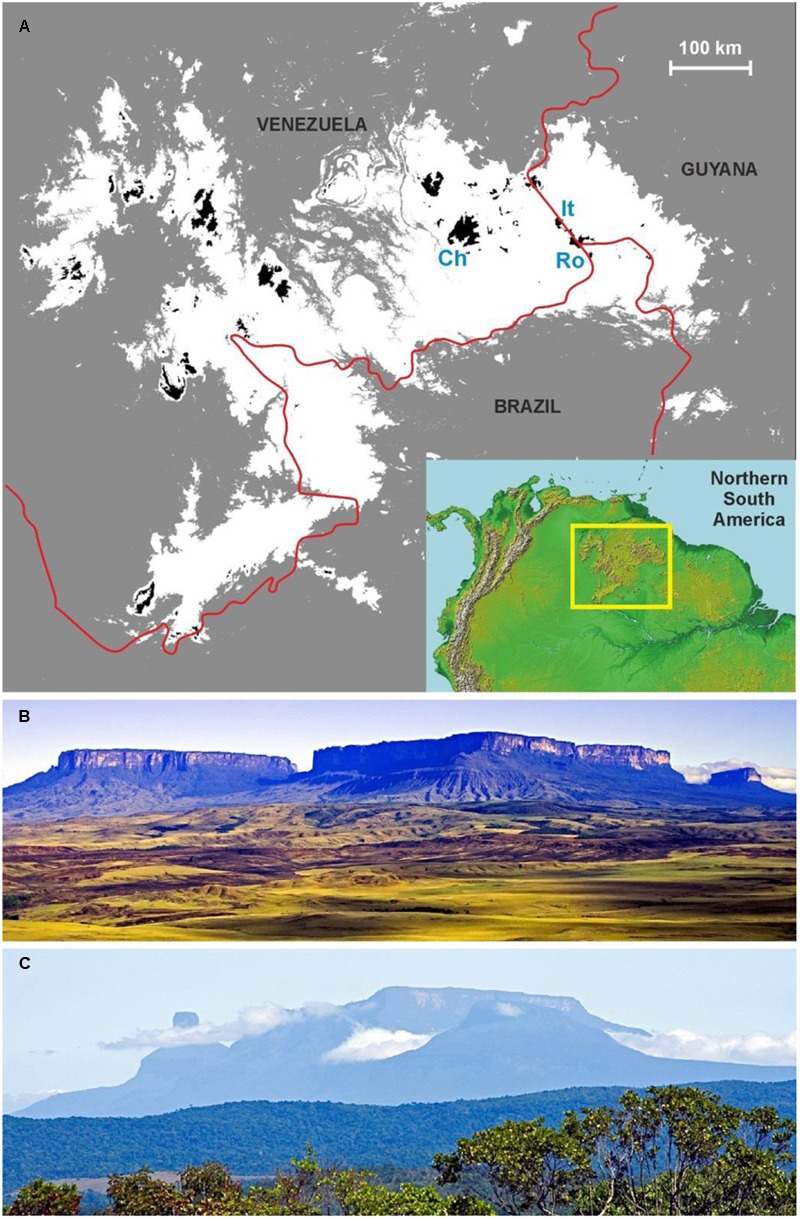
**Map of the study location and example of tepuian massifs. (A)** Map with the studied area in the geographical context of northern South America. The Amazon and Orinoco lowlands are in gray; the Guayana Highlands (GH) region in white with the GH proper (i.e., situated above 1500 m in elevation) in black. Ch – Chimantá massif, It – Ilú-Tramén massif, Ro – Roraima tepui. Red lines are the international borders. **(B)** The Roraima (right) and the Kukenán (left) tepuis. The summit of the Roraima has a surface area of 34.38 km^2^ and a maximum elevation of 2723 m, whereas the Kukenán summit has a surface area of 20.63 km^2^ and a maximum elevation of 2650 m (Huber, 1995a). **(C)** The Ilú-Tramén massif, whose summit has a surface area of 5.63 km^2^ and a maximum elevation of 2700 m (Huber, 1995a) (Photos: V. Rull).

The GH are composed of the flat summits of the ∼60 sandstone table mountains (locally called tepuis) in the neotropical region of Guayana (**Figure 1**), whose unique biotic features define the Pantepui biogeographic province (Huber, 1994; Berry et al., 1995). The total extent of these summits is ca. 6000 km^2^, and they range from 1500 to 3014 m in elevation (Huber, 1995a). This study is centered on Venezuela, the country to which most tepuis belong. The GH emerge from the surrounding lowlands as an archipelago of islands suspended in the air and are characterized by outstanding plant diversity and levels of endemism similar to those of oceanic islands (Berry and Riina, 2005; Rull, 2009b). A database built with the currently available floristic information revealed the occurrence of 2433 species (>4000 species per 10,000 km^2^), of which 618 (ca. 25%) are endemic to Pantepui (Safont et al., 2012). Considering that many tepui summits remain largely unexplored (Huber, 1995b), these numbers are extraordinary in a global context (Barthlott et al., 1999). These mountain islands are remote and mostly inaccessible; only a few summits can be reached by foot. The indigenous people inhabiting the region call these table mountains “tepuis” and consider their summits to be the home of gods, from which humans are forbidden. There are no economically profitable resources on the tepui summits, and activities, such as agriculture, livestock husbandry, forestry or mining are prevented by the particular geologic, edaphic, and biotic conditions. The only activities that have been developed in the GH are tourism and scientific exploration, and access is mainly by helicopter (Huber, 1995b). There is no tourist infrastructure on the GH (visitors should camp on the summits), so their pristinity is maintained.

The GH are protected by several designations including national parks, a Biosphere Reserve and a World Heritage Site, and the tepuis themselves were declared a natural monument in 1990 (Huber, 1995c). However, the existing framework is not sufficient to guarantee their conservation. For example, the latest environmental evaluation of the Canaima National Park, the most emblematic protected area in the region, concluded that while the conservation status of the tepuian summits is good, the level of threat is high, especially to biodiversity (Novo and Díaz, 2007). The report also identified several obstacles to a suitable conservation policy, namely, the insufficient level of funding; the scarcity, high turnover and incomplete training of the personnel responsible daily field surveillance; the enormous size of the park and the remoteness of many protected areas; the diversity of actors and stakeholders, including governmental, private and indigenous interests, and the absence of a mechanism of exchange among them; and the lack of a definite management plan for the park, although some proposals exist (Bevilacqua et al., 2009). The Guiana Shield Facility (GSF), an international conservation organization that works in the Guayana/Guiana Shield countries, considers the tepuis to be priority areas for protection (Huber and Foster, 2003; Bernard et al., 2011), but specific actions have not yet been attempted. Furthermore, many tourist activities are illegal or out of control, and the first signs of human disturbance are beginning to appear. For example, in the Roraima (**Figure 1**), the most visited tepui, vegetation trampling and other direct impacts are not uncommon, and recent studies have documented the introduction of invasive plants and the contamination of water by fecal bacteria (Safont et al., 2014; Fernández-Delgado et al., 2016). Urgent actions are needed to control this particular problem (Rull et al., 2016), which, fortunately, is an exceptional situation, and most of the tepuian summits remain virtually untouched.

## Range Shifts and Habitat Loss

Despite their general pristinity and the paucity of direct human impact, the GH vascular flora is considered to be severely threatened by climate change (Rull and Vegas-Vilarrúbia, 2006). GW is considered a major threat to mountain flora worldwide as plants may respond by shifting their altitudinal distributions, leading to changes in the diversity and composition of their communities as well as the reduction, fragmentation or loss of their habitat. An increasing number of studies show that this is already occurring and warn of the potential threat of GW to summit species and communities (e.g., Thuiller et al., 2005; Pauli et al., 2007, 2012; Colwell et al., 2008; Kelly and Goulden, 2008; Lenoir et al., 2008; Grabherr et al., 2010; Kreyling et al., 2010; Walther, 2010; Dirnböck et al., 2011; Engler et al., 2011; Feeley et al., 2011; Sheldon et al., 2011; Gottfried et al., 2012; Jump et al., 2012). Tropical mountains are of particular concern because of their high biodiversity and levels of endemism (Davis et al., 1997; Myers et al., 2000; Malcom et al., 2006; Laurence et al., 2011; Nogué et al., 2013). Indeed, the extinction of an endemic species is a global extinction.

In the GH, the risk of extinction by habitat loss due to the projected GW in this century has been addressed using the altitudinal range shift (ARS) method (Rull and Vegas-Vilarrúbia, 2006) and climate envelope distribution models (CEDM) (Rödder et al., 2010). Both methods use geographic information systems (GIS) techniques and have been applied to estimate the expected habitat reduction for Pantepui species assuming that these species will respond to GW by upward migration at rates similar to that of the warming. Using the ARS method, the present potential distribution area of the Pantepui habitat was first plotted in a digital elevation model (DEM), and its reduction was simulated for a given increase in temperature and an average adiabatic lapse rate of -0.6°C per 100 m of elevation, which is characteristic of the Guayana region (Huber, 1995a). The reduction in the number of species was then calculated using the previously developed species-area relationship for the GH based on empirical data (Nogué et al., 2009). Simulations were run for the more optimistic (B1) and the more pessimistic (A2) scenarios in the third IPCC assessment report (Houghton et al., 2001), which predicted warmings of 2.0 and 4.0°C, respectively, for northern South America by the end of this century. Under these conditions, the ARS method yielded estimates of 1700 (B1)–1824 (A2) species (75–79% of the total) at risk of habitat loss, of which 209 (B1)–406 (A2) (28–54%) were endemics. An individual species-by-species analysis based on their specific estimated reductions in habitat confirmed that 166–343 endemic species (22–46%) were threatened by habitat loss (Nogué et al., 2009). All of these species are listed in Nogué et al. (2009). In addition, virtually all of the analyzed species showed a fragmented potential distribution by the end of the century regardless of the IPCC scenario (Nogué et al., 2009). More sophisticated CEDM produced similar results that supported previous ARS estimates (Rödder et al., 2010), and the CEDM-generated maps of the potential habitat by the end of this century were nearly identical to those obtained by ARS. However, CEDM maps were not used to estimate the number of species at risk of habitat loss.

The ARS was re-run under the new predictions of the fourth IPCC assessment report (Solomon et al., 2007), but the estimated number of endangered endemic species did not change significantly (Safont et al., 2012). However, this report considered a three-step warming: (I) 1.0°C (for both B1 and A2) by 2011–2030, (II) 2.0°C (B1) to 2.5°C (A2) by 20146–2065, and (III) 2.5 (B1) to 4.0°C (A2) by 2080–2099, which facilitated a preliminary hierarchization of the endangered species. The ARS analysis was individually run for the three warming phases, and the 35 endemic species (6%) losing their potential habitat at stage I were considered the most threatened, followed by the 140 (B1)–184 (A2) (23–30%) predicted to lose their habitat at stage II and, finally, the 184 (B1)–307 (A2) (30–50%) that will be at risk of habitat loss by the end of the century (stage III). An exhaustive list of these species is provided by Safont et al. (2012).

## Conservation Strategies

The above species-level risk analysis was complemented by defining conservation priority categories using an index called the environmental impact value (EIV) for each species, which considered the taxonomic level of endemism (i.e., family, genus, species), the degree of endemism (i.e., local tepui endemism, general Pantepui endemism, tepuian district endemism), the characteristic of keystone species (when a given species is fundamental to the existence of its community), and the geographical and altitudinal ranges of the species (Safont et al., 2012). Prioritizing species according to extinction threat may be a more cost-effective way to invest conservation resources than comprehensive plans intended to preserve all species at once or only the most charismatic (Pärtel et al., 2005; Jiménez-Alfaro et al., 2010). The EIV defined 10 (B1)–13 (A1) priority conservation categories; species deserving immediate conservation actions were those estimated to lose their habitat during stage I with the highest EIV scores, which corresponded to priority categories 1–3 for both the B1 and A2 scenarios. A complete list of all priority categories is provided in Safont et al. (2012).

**FIGURE 2:**
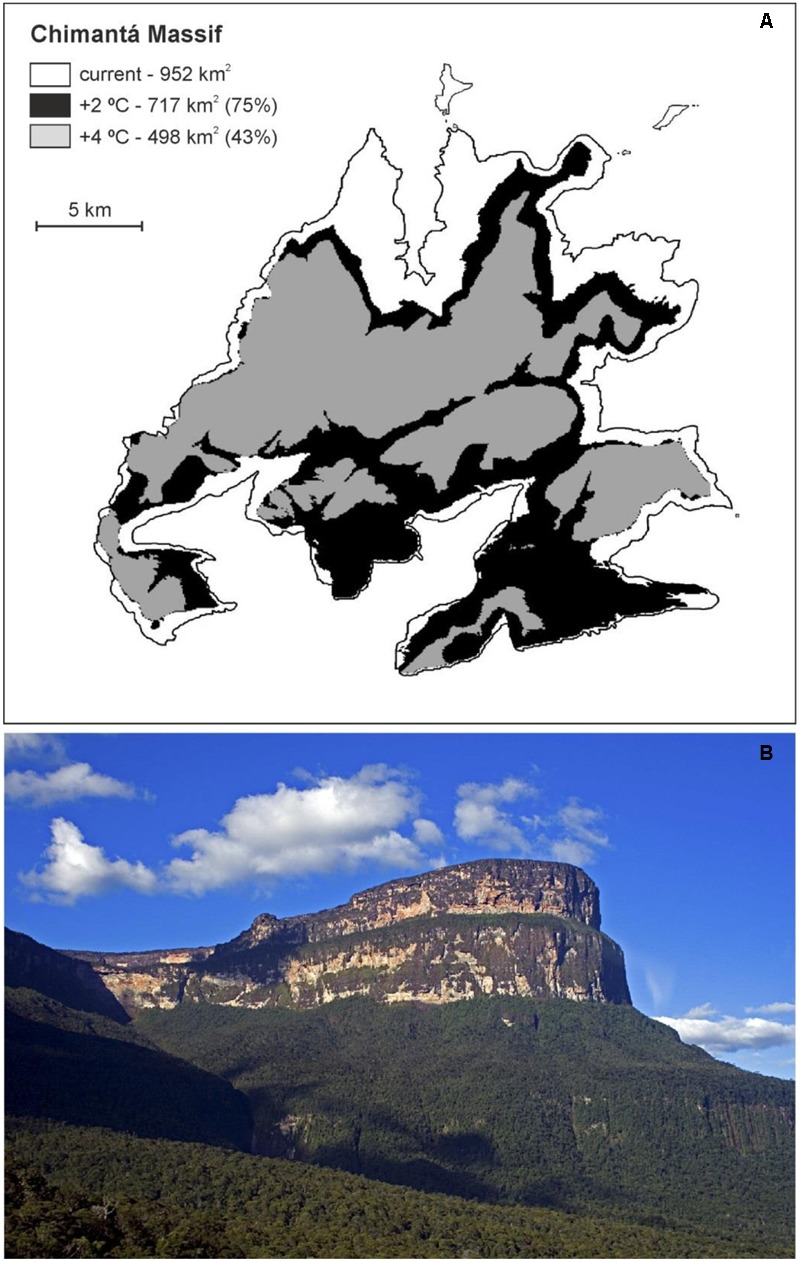
**Pantepui habitat reduction (in %) on the Chimantá massif. (A)** Geographic information systems (GIS)-generated map of the summit contour of the Chimantá massif (>1500 m in elevation), indicating the Pantepui habitat area for warmings of 2°C (black area) and 4°C (gray area). **(B)** NE cliffs of the Chimantá massif (Photo: V. Rull).

In general, the best strategy for long-term biodiversity conservation is considered to be *in situ* conservation, notably the enhancement of the degree of protection or designation of new protected areas (Frankel et al., 1995; Primack, 2002). However, the danger of habitat loss due to GW in the absence of direct human impacts, as is the case in the GH, cannot be addressed with *in situ* practices alone, so *ex situ* measures seem to be required to preserve the biodiversity and ecosystems of the Pantepui. It has been estimated that, by the end of this century, the Pantepui area will be reduced by >80% and that the suitable habitat for Pantepui species will disappear from more than 35 (58%) tepuis. The largest remaining patch will be located in the Chimantá massif (**Figure 2**), which will represent nearly the half of the total remaining Pantepui area (Vegas-Vilarrúbia et al., 2012). This patch can be considered a potential refuge for future GH flora, which would contain resistant species from lower altitudes and other species that will eventually persist in microrefugia (Rull, 2009a). Maps of the potential Pantepui area remaining by 2100, in which eventual *in situ* actions should be concentrated, are available in Vegas-Vilarrúbia et al. (2012).

Among the available *ex situ* techniques, germplasm banks, living plant collections and managed relocation have been discussed (Rull and Vegas-Vilarrúbia, 2006; Nogué et al., 2009; Safont et al., 2012). Germplasm preservation seems to be especially feasible as it will allow for the restoration of species when needed, provided the suitable environmental conditions are present. Classical seed collection and banking as well as the creation of botanical gardens have already been suggested as suitable methods for preserving endangered species for restoration purposes. However, in these procedures, special care should be taken to preserve genetic diversity as much as possible (Vitt et al., 2010; Hardwick et al., 2011). Managed relocation is a more controversial approach due to the likelihood of unexpected ecological consequences in recipient ecosystems (Hunter, 2007; McLachlan et al., 2007; Davidson and Simkanin, 2008; Hoegh-Guldberg et al., 2008; Ricciardi and Simberloff, 2009; Seddon et al., 2009), so to minimize potential ecological impacts, relocated species may be restricted to selected areas along an elevational transect according to their individual requirements and isolated from autochthonous communities, i.e., in artificial microrefugia. The northern Andes have been proposed as a possible recipient area as species would be allowed to migrate upwards due to the occurrence of suitable terrain above 3000 m in elevation (Rull et al., 2009). Potential relocations must be informed by carefully planned ecological studies to ensure that the creation of novel ecosystems for stressed populations do not damage the recipient ecosystem (Safont et al., 2012).

## Some Problems and New Perspectives

There are several issues with the habitat loss estimations that have been performed in the GH to date. First, environmental factors other than temperature could act as drivers of species range shifts. Second, the migration capacity of species could be limited by the lack of suitable substrates or topographical barriers. Third, the estimation methods that have been used assume that all species will respond to warming by migrating upwards at the same rates. Therefore, the available potential habitat loss estimates should be considered preliminary and as a first step toward more realistic projections that consider all relevant environmental factors as well as the ecological features of the species of concern that could affect their individual responses to climate change.

In terms of climatic factors other than temperature, precipitation intensity and distribution is predicted to undergo significant shifts worldwide during this century, but such changes seem to be of relatively little relevance to the studied region. On the one hand, the 0–5% reduction in mean annual rainfall forecasted by the IPCC by the end of this century (Solomon et al., 2007; Stocker et al., 2013) is almost negligible considering the perhumid climate of the tepui summits, where precipitation ranges from 2500 to 4000 mm per year with little seasonal variation (Huber, 1995a). On the other hand, temperature has been recognized as the main factor controlling the altitudinal distribution of vascular plant species in Pantepui (Huber, 1995a) as in many other areas (e.g., Guisan and Theurillat, 2000; Thuiller et al., 2005). The projected increase in the concentration of atmospheric CO_2_ is another factor that should be considered, and evaluating its effects requires detailed eco-physiological studies of selected species, such as those in the priority categories mentioned above. Unfortunately, such studies are not available and are difficult to perform due to logistic and bureaucratic constraints (see below). The influence of local topographical and edaphic factors (e.g., slope orientation, substrate availability, microclimatic conditions, and vegetation structure) might be relevant to the control of altitudinal plant migration and could be understood with intensive fieldwork and the use of GIS tools. Again, acquiring detailed knowledge of these aspects has been hindered by the difficulty of developing ecological studies atop the tepuis, but the need for these types of studies is indisputable and methods that are applicable globally, such as those developed by the GLORIA (Global Observatory Research Initiative in Alpine environments) project (Pauli et al., 2005) have been encouraged (Rull et al., 2009).

Increasing the autoecological knowledge of the potential responses of threatened plant species is imperative. Current predictive models for the GH assume homogeneous responses, but this should be considered approximative as different species may respond to warming in different ways. Species with higher phenotypic plasticity might be able to tolerate warming by accommodating their morphological, physiological or life history traits to changing climates. Examples include changing photosynthesis rates and growth in response to increasing atmospheric CO_2_ concentrations or modifying phenological features, such as growth and flowering times and duration, in response to changing meteorological seasonality (Ainsworth and Rogers, 2007; Gunderson et al., 2010; Medeiros and Ward, 2013; Liancourt et al., 2015). The combined effects of warming, water stress and elevated CO_2_ should also be taken into account (e.g., Xu and Zhou, 2006; Xu et al., 2014, 2016). At the ecosystem level, such changes could influence the resilience of species to climate change and, consequently, their competitive ability, symbiotic interactions and fitness, which may affect community composition and ecological functioning (Kimball et al., 2012). According to Becklin et al. (2016), phenotypic plasticity may alleviate the effects of climate change but may not guarantee long-term persistence, especially if environmental change exceeds the variability that species have experienced historically. The species that are unable to accommodate to GW in this way, should adapt, in an evolutionary sense, to the new environmental conditions or migrate; otherwise, they will become extinct *in situ*. The potential occurrence of rapid evolutionary adaptation, i.e., genetic change, to GW is a controversial issue (Merilä, 2012) that remains difficult to demonstrate (Franks et al., 2007). A necessary condition for species to undergo rapid evolutionary change in response to climate change is sufficient genetic variation in the traits under selection pressure (Becklin et al., 2016), but such knowledge of the GH does not exist for the same reasons mentioned above and should be urgently addressed in light of the priority categories established for the more threatened species (Safont et al., 2012).

In addition to the remoteness and low accessibility of the GH, a recurrent obstacle to a sound assessment of the plant species and communities threatened with extinction by habitat loss is the difficulty obtaining fieldwork and sampling permits. This problem was first noted several years ago (Rull and Vegas-Vilarrúbia, 2008; Rull et al., 2008), but the situation has not changed. The lack of sufficient environmental management tools (Novo and Díaz, 2007) and the difficulty of establishing effective control mechanisms in a region as vast as the GH are serious challenges to adequate protection policies (Rull et al., 2016). Currently, visits to most of the tepuis are prohibited, and scientific surveys, especially those related to genetic studies, are subjected to serious restrictions to prevent biopiracy (Rull and Vegas-Vilarrúbia, 2008). These restrictions should be relaxed to allow a proper appraisal of the potential effect of GW on GH species and their ecosystems. Current estimates based on the available databases and methods, such as ARS and CDEM, are all that can currently be accomplished. Without new and more detailed ecological and physiological studies of selected GH plants and ecosystems, the background information required for optimizing conservation practices will remain unknown. In these conditions, the use of remote sensing techniques would be useful, especially those involving radiation measurements related to ecophysiological features such as energy balance or photosynthesis (Jones et al., 2003). Such measurements might be done using conventional satellite imagery or newly developed techniques as for example Unmanned Aerial Vehicles (UAV), also known as drones (Salamí et al., 2014).

## Author Contributions

VR and TV contributed equally to the ideas expressed here. VR wrote the manuscript and TV provided additional insights.

## Conflict of Interest Statement

The authors declare that the research was conducted in the absence of any commercial or financial relationships that could be construed as a potential conflict of interest.
